# Prevalence and risk factors of seizure in children with acute bacterial meningitis: updating previous evidence using an epidemiological design

**DOI:** 10.22037/ijcn.v15i2.22250

**Published:** 2021

**Authors:** Alireza ATAEI NAKHAEI, Elham BAKHTIARI, Sara GHAHREMANI, Javad AKHONDIAN, Mohammad Saeed SASAN, Malihe MOVAHED, Mohammad Hassan AELAMI

**Affiliations:** 1 Department of Pediatrics, Faculty of Medicine, Mashhad University of Medical Sciences, Mashhad, Iran; 2 Eye Research Center, Mashhad University of Medical Sciences, Mashhad, Iran; 3 Department of Pediatric Neurology, Faculty of Medicine, Mashhad University of Medical Sciences, Mashhad, Iran; 4 Infection Control &Hand Hygiene Research Center, Mashhad University of Medical Sciences, Mashhad, Iran

**Keywords:** Meningitis, Child, Seizure, Iran

## Abstract

**Objective:**

The current study aimed to investigate the prevalence and risk factors of seizure in acute bacterial meningitis.

**Materials & Methods:**

In the present study, a total of 180 children (age range, 2 months to 14 years) with acute bacterial meningitis, were separated into two groups based on the diagnosis of seizure. The study was conducted in Mashhad (Iran) from 2002 to 2016.

**Results:**

Seizure occurred in 37.4% of children with bacterial meningitis. *Streptococcus pneumonia* (*S. pneumonia*) was the most common organism. Most of the children with seizures (53.7%) had more than one episode. Also, 35% of patients had neurologic complications. Complications were more related to the seizure occurrence, the number of episodes, prolonged seizure, and being younger than 12 months. Age categories of less than 1 year and 1-5 year were associated with increased risk of seizure (odds ratio: 4.33 and 6.54, respectively). The more episode of seizure was associated with more complications (odds ratio: 6.33).

**Conclusion:**

The prevalence of seizures in acute bacterial meningitis was 37.4%. Besides, the seizure was associated with more complications. Hence, timely diagnosis and treatment of bacterial meningitis are necessary for preventing future consequences.

## Introduction

Acute bacterial meningitis (ABM) is a life-threatening infection with high morbidity and mortality in neonates and young children (1, 2). Every year it claims more than 171000 life worldwide (3). The mortality rate of bacterial meningitis ranges from 2 to 30% (3). Also, 10-20% of survived cases suffer from epilepsy and mental retardation (1). The mortality rate of ABM depends on age, pathogen, and geographic location (3). Furthermore, its epidemiology has changed considerably in the countries that adopted the conjugate vaccines against *Haemophilus influenza* type b, *Neisseria meningitides*, and *S. pneumoniae* (4). The ABM carries a higher burden in developing countries (4). Seizure is a common and unwelcome outcome of bacterial meningitis. The incidence rate of seizure in patients who suffer from ABM varies from 25 to 50% (5-7). Our literature review revealed that no recent study has been conducted on this issue in Iran, as a developing country. Hence, following a retrospective design (2002 to 2016) the current study aimed to evaluate the prevalence and risk factors of seizure in infants and children with acute bacterial meningitis in Iran.

## Patients and methods

The current clinical study was conducted retrospectively in Imam Reza hospital, as a referral healthcare center, affiliated to the Mashhad University of Medical Sciences, Mashhad (Iran) from 2002 to 2016. The study is approved by the Ethics Committee of the Mashhad University Medical Sciences (IR.MUMS.FM.REC.1394.245). A total of 180 children (age range, 2 months to 14 years) with acute bacterial meningitis, were separated into two groups based on the diagnosis of seizure. The study was conducted in Mashhad (Iran) from 2002 to 2016. The diagnosis of acute bacterial meningitis was documented based on the World Health Organization (WHO) protocol: Bacterial meningitis is defined by fever, headache, and one of the following signs: neck stiffness, altered consciousness, or other meningeal signs. Bacterial meningitis can be confirmed by isolation of a bacterial pathogen from a normally sterile clinical specimen such as CSF or identification of a bacterial antigen in normally sterile fluids or gram stain results. 

**Sample size**

Following the enumeration method, the sample size was estimated at 180 subjects.

**Analysis**

Data were analyzed using SPSS version 16 (SPSS Institute, Inc., Chicago, IL, USA). All experimental values are presented as Mean ± standard deviation (SD). The t-test was used to compare the study groups. The association between qualitative variables was calculated by the chi-square test. Risk factors were calculated according to the logistic regression model. Statistical significance was considered when p-value<0.05. 

## Results

***Demographic***

Of 180 participants, the seizure was observed in 67 patients (37.4%). Also, 111 patients (61.67%) were male and 69 (38.33%) were female. Besides, 55 patients with seizure (67.2%) and 66 without seizure (58.9%) were male, with no significant difference concerning the variable of gender (p=0.2). The mean age of participants was 36.25±42.51 months, with a median of 13 months. The youngest and oldest participants were 2 and 158 months old. Forty-six patients with seizure (68.7%) and 36 without seizure (32.1%) were aged less than 1 year, with a significant difference (p=0.001). Six patients (3.3%), including 4 with seizure and 2 without seizure, died during the study period. The demographic characteristics of participants are presented in Table 1. 

***Organisms***

According to smear and CSF culture, *S. pneumoniae* was found in 16 (23.9%) patients with seizure and 25 (22.7%) without seizure. *H. influenza* type b was found in 4 (6%) and 12 (10.9%) patients with and without seizure, respectively. Forty-four (65.7%) and 67 patients (60.9%) with and without seizure had negative results, respectively. There was no significant difference between the study groups concerning the responsible organism (p=0.54). Also, the type of the organism was not different in patients with and without seizure. The results are presented in Table 2. 

***Seizure characteristics***

Of 180 eligible patients, 67 (37.4%) had at least one episode of seizure. Type of seizure was generalized in 53 patients (79.1%). Also, 63 patients (35%) experienced neurologic complications including hydrocephaly (n=20; 31.7%), subdural effusion (n=14; 22.2%), and hearing impairment (n=11; 17%). Among patients with generalized seizure, 28 (52.8%) developed at least one complication. Of 67 patients with seizure, 36 (53.7%) had more than one episode of seizure. The prolonged seizure was observed in 17 (15.2%) patients. Seizure occurred before hospitalization in 23 patients (34.3%) and on the first day of admission in 22 patients (32.8%). Seizure characteristics are presented in Table3. There was a significant difference concerning the seizure occurrence, the number of seizure episodes, prolonged seizure, and being younger than 1 year old (p=0.002, 0.002, 0.007, and 0.0001, respectively). Complications were not significant according to the type of seizure, time of first seizure episode, gender, and mortality (p>0.05). 

***Laboratory characteristics***

Among patients with seizure, 45 (69.2%) had CSF glucose less than 40 mg/dl. For patients without seizure, 70 cases (63.1%) had CSF glucose less than 40 mg/dl. The difference was not significant (p=0.4). Thirty patients (44.8%) with seizure had more than 1000 leukocytes in the CSF sample but in patients without seizure. In addition, 63 patients (56.2%) had more than 1000 leukocytes in the CSF sample; However, the difference was not significant (p=0.2). 

Among patients with seizure, 58 (89.2%) had the CSF protein more than 40 mg/dl. In patients without seizure, 103 (92.8%) had the CSF protein more than 40 mg/dl (p=0.4). The results are presented in Table 4. 

***Risk factors***

According to the results of the logistic regression, age less than 1 year and the age group of 1 to 5 years old had a significant association with seizure occurrence (OR= 4.33; CI: 4.33-42.22, P=0.0001; and OR=6.54: CI: 1.95-21.95, P=0.002, respectively). Again, according to the logistic regression, the number of seizure episodes had a significant association with complications incidence (OR= 6.33; CI: 1.77-22.63, p=0.005). Data are presented in Tables 5 and 6.

## Discussion

In the present study, 180 children with acute bacterial meningitis referred to the Imam Reza hospital for the period of 2002 to 2016 were recruited. The prevalence of seizure was 37.4%. Nearly 79% of patients had a generalized seizure and 15.2% had a prolonged seizure. For 34.3% of patients, the seizure occurred before the hospitalization. *S. pneumoniae* was the most common organism detected among the patients with or without seizure. Complication occurred in 35% of patients with the most frequency of hydrocephaly (31.7%). There was a significant difference between patients with and without seizure in the rate of complications. Several studies in different countries have investigated the rate of bacterial meningitis according to CSF cultures and reported that the rate of seizure associated with meningitis and the type of responsible organism depend on geographical area (5, 8-10). 

According to the findings, the prevalence of seizure was 37.4%. Unfortunately, the rate of seizure in bacterial meningitis is more in developing countries. In a study in Brazil by Gomes, the rate of seizure in bacterial meningitis is reported at 38.1%. Or another study in Taiwan reported a rate of 47%, while rates of 27% and 31% are reported for the USA in 1985 and 1990 (5, 8, 9, 11). Also, according to the findings, the rate of mortality was 3.3%, which is in line with several studies that reported a prevalence of 3.8 to 12% (12-15). 

In our study, being younger than one-year-old and age group of 1 to 5 years were associated with increased risk of seizure incidence in children with ABM; [OR= 4.33; 95% CI: 4.33-42.22]. For those younger than one-year-old and (OR=6.54; 95% CI: 1.95-21.95). For those aged one and five years old. In a study on 270 children with confirmed bacterial meningitis in Brazil, Corrêa-Lima et al. investigated the occurrence of in-hospital symptomatic seizures. Age less than 2 years (OR = 0.97; CI: 0.97-0.98), pneumococcal etiology (OR= 4.55; CI: 1.88-11.0), and altered mental status (OR= 3.47; CI=1.66-7.26) were associated with increased risk of seizure (16). The present study showed that patients with meningitis and seizure had significantly higher complications. Also. some other studies reported a significant association between seizure and complications like arthritis, subdural effusion, and hydrocephaly, and hearing impairment, which is in line with the findings of the present results (12, 17, 18). According to the findings of the present, study there was no significant difference concerning the seizure occurrence between boys and girls, while some studies reported a significant difference (19). 

 In this study, the frequency of seizure episodes was accompanied by experiencing more complications in patients with ABM (OR=6.33; **95% CI: **1.77-22.63). Corrêa-Lima et al. reported that the mortality rate was higher among patients with intra-hospital epileptic seizures compared to those without this health problem (25/67 [37.3%] vs 9/203 [4.43%], P < .001] (16). 

Previous studies suggested that *S. pneumoniae *and *N. meningitidis *are the most common organisms causing ABM. In a study by Chinchankar in India on the common responsible organism for bacterial meningitis, it has been reported that *S. pneumoniae* and *H. influenza* are the most common responsible organisms (39% and 26%, respectively) (20). In another study in Greece (2011), *N. meningitides* is recognized as a common organism in meningitis, with a rate of 63% (12). The findings of the present study are consistent with previous reports. In Iran, vaccination against *H. influenza* was started in 2014. Our findings indicated that the rate of *H. influenza* infection in children with meningitis was 11.4% before vaccination, which decreased to 3.2% after vaccination, which in turn indicates the critical role of vaccination in the prevention or decrement of bacterial meningitis. 

**Table1:** Demographic characteristics of 180 patients with acute bacterial meningitis

**Variable**	**with seizure**	**without seizure**	**Total population**	**P value**
**Age ** Less than 1 year 1-5 years More than 5 years	46 (68.7%) 17 (25.4%) 4 (6%)	36 (32.1%) 32 (28.6%) 44 (39.3%)	82 (45.8%) 49 (27.2%) 48 (26.7%)	0.0001*
**Gender** Male female	45 (67.2%) 22 (32.8%)	66 (58.9%) 46 (41.1%)	111 (61.7%) 69 (38.3%)	0.27

*Significant in chi square

**Table2:** Responsible organisms detected by smear and CSF culture in 180 patients with acute bacterial meningitis

**Variable**	**Streptococcus pneumoniae**	**Haemophilus influenza type b**	**Neisseria meningitides**	**Other oraginisms**	**No organism**	**Total**	**P value**
**With seizure**	16 (23.9%)	4 (6%)	0	3 (4.5%)	44 (65.7%)	67 (100%)	0.54*
**Without seizure**	25 (22.7%)	12 (10.9%)	3 (2.7%)	3 (2.7%)	67 (60.9%)	110 (100%)	
**Total**	41 (23.2%)	16 (9%)	3 91.7%)	6 (3.4%)	111 (62.7%)	177 (100%)	

* Chi square

**Table 3:** Seizure characteristics of patients with acute bacterial meningitis

**Seizure characteristics**	**Frequency (n)**	**Percent (%)**
**Type of seizure**		
Focal Generalized Both **Total**	12 53 2 67	17.9 79.1 3 100
**Number of episodes**		
Once More than once **Total**	31 36 67	46.3 53.7 100
**Complication**		
Hydrocephaly Subdural effusion Hearing impairment Hydrocephaly and hearing impairment Paresis Cerebritis Subdural effusion and paresis Subdural effusion and cerebritis Visual impairment Hearing and visual impairment **Total**	20 14 11 7 5 2 1 1 1 1 63	31.7 22.2 17 11.1 7.9 3.2 1.6 1.6 1.6 1.6 100
**Status seizure** **No status seizure** **Total **	17 50 67	15.2 84.8 100
**Time of Seizure **		
Before the third day After the third day **Total**	52 15 67	77.6 22.4 100

**Table4:** Laboratory characteristics in 180 patients with acute bacterial seizure

**variable**	**With seizure**	**Without seizure**	**P value**
CSF glucose <40 mg/dl	45 (69.2%)	70 (63.1%)	0.4
Leukocyte>1000 cell/mm^3^ in CSF sample	30 (44.8%0	63 (56.2%)	0.2
CSF protein > 40 mg/dl	58 (89.2%)	103 (92.8%)	0.41

**Table5:** Odds ratio for involved variables in seizure incidence in patients with acute bacterial meningitis

**variable**	**Odds ratio**	**CI (95%)**	**P value**
**Age<1 year**	**4.33**	**4.33-42.22**	**0.0001**
**Age between 1-5 year**	**6.54**	**1.95-21.95**	**0.002**
Leukocytosis<100	1.78	0.6-5.2	0.29
Leukocytosis between 100-1000	1.36	0.64-2.88	0.41
Negative leukocytosis	3.29	0.06-171.35	0.55

**Table6:** Odds ratio for involved variables in complication occurrence in patients with acute bacterial meningitis

**Variable**	**Odds ratio**	**CI (95%)**	**P value**
**Number of seizure**	**6.33**	**1.77-22.63**	**0.005**
prolonged seizure	1.43	0.35-5.74	0.6
Gender	2.5	0.73-7.61	0.14

## In Conclusion

To conclude, the prevalence of seizure in patients with acute bacterial meningitis was 37.4%. Because of the significant association between seizure and complication, it is necessary to pay more attention to the exact and on-time diagnosis of acute bacterial meningitis, as it can decrease both hospitalization and cost of treatment and is associated with increased quality of life.

According to the findings, we suggest routine administration of *S. pneumoniae* vaccine for children aged 2, 4, and 6 months as well as those aged 12-18 months in Iran. 

## Author’s contribution

Alireza Ataei Nakhaei, Mohammad Hasan Aelami, Malihe Movahed: conception or design 

Elham Bakhtiari, Mohammad Saeed Sasan, Javad Akhondian, Sara Ghahremani: acquisition, analysis 

 Elham Bakhtiari, Alireza Ataei Nakhaei, Mohammad Hasan Aelami, Malihe Movahed, Mohammad Saeed Sasan, Javad Akhondian, Sara Ghahremani: drafting the work 

Elham Bakhtiari, Alireza Ataei Nakhaei, Mohammad Hasan Aelami, Malihe Movahed, Mohammad Saeed Sasan, Javad Akhondian, Sara Ghahremani: final approval

## Conflict of interest

The authors declare that there is no conflict of interest.
